# Prevalence of selected intestinal protozoan infections in marginalized rural communities in Palestine

**DOI:** 10.1186/s12889-019-8024-2

**Published:** 2019-12-11

**Authors:** Amer Al-Jawabreh, Suheir Ereqat, Kamal Dumaidi, Hanan Al-Jawabreh, Ziad Abdeen, Abdelmajeed Nasereddin

**Affiliations:** 1 Department of Medical laboratory Sciences, Faculty of Allied Health Sciences, Arab American University, Jenin, Palestine; 2 Al-Quds Public Health Society, Jerusalem, Palestine; 3 Leishmaniases Research Unit, Jericho, Palestine; 4 Biochemistry and Molecular Biology Department, Faculty of Medicine, Al-Quds University, Abu Deis, Jerusalem, Palestine; 5 Al-Quds Nutrition and Health Research Institute, Al-Quds University, East Jerusalem, Palestine

**Keywords:** Intestinal parasitic infection, Palestine-West Bank, PCR, Real-time PCR, *Giardia lamblia*

## Abstract

**Background:**

Intestinal parasitic infections are common in rural areas with poor infrastructure and low socioeconomic status. The aim of this study was to estimate the prevalence of selected parasitic infections in marginalized rural areas in the northern part of the Palestinian West Bank Region, using conventional and PCR-based methods, and also to assess risk predictors of infection.

**Methods:**

A cross-sectional study was conducted on 104 individuals from three rural villages in the Jordan Valley. Stool samples were collected and examined by a battery of tests that included microscopy of wet fecal samples in normal saline with iodine, concentration by ethyl acetate sedimentation and also by zinc sulfate floatation, a conventional PCR and a real-time PCR (qPCR). Risk factors were assessed that included demographic, socioeconomic, and behavioral characteristics. Data on method performance was analyzed by kappa-statistic, Cochrane’s Q, and McNemar post hoc test. Mid-P exact test and odds ratio were used to discern association between outcome and risk predictors.

**Results:**

The overall prevalence of intestinal parasitic infections was 48% (49/102). The predominant parasites were *Giardia lamblia* at 37% (37/102) and *Hymenolepis nana* at 9% (9/102). To concentrate cysts and eggs, sedimentation can be used as an alternative to floatation with a loss of 1% of positive cases. The methods employing PCRs proved crucial as it increased the detected infection rate of *G. lamblia* approximately three-fold from 13% by the conventional methods to 37% by the qPCR. Multiple infections were present in 13% (13/102) of the study group, which included double (10%) and triple (3%) infections. Regarding the genus *Entamoeba*, *E. dispar* and *E. coli* were detected at rates of 2 and 8%, respectively. While none of the individuals were infected with the pathogenic *E. histolytica*, *E. nana* (4%) was detected for the first time in the area. Age was a risk predictor for infection (OR = 2.61, CI 95% 1.05–6.45, *P* = 0.038).

**Conclusions:**

The increased prevalence of intestinal parasitic infections in children in marginalized rural areas in Palestine is worrying. The addition of PCR-based methods is important for the diagnosis of such infections as, with cautious interpretation, it increases proficiency and overcomes underestimation and misdiagnosis of cases. Control measures including education on personal hygiene and environmental sanitation, should be introduced to reduce the prevalence of the intestinal parasites and, thus, the infections they cause in this and other areas.

## Background

The diseases caused by intestinal parasites are food- and water-borne diseases transmitted via the fecal-oral route. Typically, they affect vulnerable marginalized groups such as children below the age of five and people living in rural areas of developing countries [1–4]. According to World Health Organization (WHO) estimates, the most common intestinal protozoa are *Giardia lamblia, Entamoeba histolytica* and *Cryptosporidium spp* [5], and in 2010 the median global burdens of disease (GBD) for these parasitic species were 0.17 million, 0.5 million, and 2 million DALYs, respectively. The median numbers of cases of giardiasis, amoebiasis, and cryptosporidiosis were 184 million, 104 million, and 64 million, respectively, and are considered underestimates. While no deaths were reported globally for giardiasis in 2010, *E. histolytica* caused 5450 and *Cryptosporidium spp* 27,553 [3, 5].

In the 1940s, the types of parasites reported for the Palestinian population included *Ascaris lumbricoides* (62%), *E. histolytica* (34%), *Trichuris trichura* (19%), *G. lamblia* (10%), *Entamoeba coli* (9%), *Trichomonas vaginalis* (3%), *Taenia saginata* (34%), *Taenia solium* (< 1%), *Ancylostoma duodenale* (< 1%), and *Hymenolepis nana* (< 1%) [6]. Studies done from 1981 to 2014 revealed similar parasite profiles and also *Cryptosporidium* but with varying degrees of prevalence, depending on the location of study and detection methods employed [1, 7–12]*.* From the year 2000, the Palestinian Ministry of Health has reported intestinal parasitic infections annually, which has included giardiasis, ascariasis, enterobiasis, strongyloidiasis, and amebiasis [13]. Over the same time period, the Israeli Health Authority has reported giardiasis and cryptosporidiosis as being the main intestinal parasitic diseases in Israel [14].. In neighbouring Jordan, which shares similar demographics, parasitic diseases with amebiasis are officially notifiable [15], and local studies have revealed the presence of *Blastocystis hominis, Giardia intestinalis, Entamoeba coli, Entamoeba histolytica, Endolimax nana, Hymenolepis nana, Enterobius vermicularis, Ascaris lumbricoides, Schistosoma mansoni, hookworms, Trichuris trichiura, Taenia saginata, Cyclospora cayetanensis and Cryptosporidium spp* [16–19]. The determinants of parasitic infections include age, overcrowding and personal hygiene practices such as hand washing, wearing of shoes, and defecation out in the open. Other risk factors include family size, level of education of the head of household, seasonality, source of drinking water, family income, and cleanliness of home [1, 8, 9, 20–22]. As estimation of prevalence is a prerequisite to develop control measures, the objective of this study was to determine the prevalence of intestinal parasitic infection in rural marginalized villages in the Jordan Valley in Palestine and to assess the risk factors associated with such infections. In doing this, molecular biological methods were employed for greater refinement in exposing infections.

## Methods

### Study area

This cross-sectional study was done in 2015–2016 and targeted three adjacent villages, Nassarieh, Beit-Hassan, and Al Aqrabaneieh, in the Palestinian part of the Jordan Valley located 50 km north of Jericho and 15 km west of Nablus. The populations of An-Nassariya, Beit-Hassan, and Al ‘Aqrabaniya numbered 1923, 1360, and 1215 inhabitants, respectively [23], most of whom were active farmers and livestock breeders. The villages stand on the foothills east of Nablus, approximately, at an latitude of 32.2437782287598 north and longitude of 35.391918182373 east (Epi Info version 7.2.2.2) and an elevation ranging from -40 m below sea level to 20 m above sea level.

### Questionnaire

The 104 individuals surveyed and examined parasitologically were selected randomly by knocking on the doors of houses without prior arrangement. After obtaining informed consent, members of households were interviewed by filling in a pre-tested questionnaire that took 15 min to complete. Four previously trained personnel carried out the interviews. The questionnaire solicited: demographic data such as age, sex, and place of residence; socioeconomic status (SES) data that included level of education, occupation and income. The questionnaire also questioned personal hygienic behavior such as hand washing as well as eating and drinking habits.

### Fecal samples

The head of each household was given a clean, labeled, wide-mouth screw cap container for each family member and thereby 102 early-morning fecal specimens were collected and transported to the diagnostic laboratory within one hour. Each fecal specimen was divided into three parts. Approximately 2 g of each specimen were transferred into 2 ml microcentrifuge tube and stored at − 20 °C for DNA analysis. The second part was used in making the wet preparation for microscopical examination. The third part was mixed with 10% formalin preservative at a ratio of 1:3 of specimen to preservative and stored for examination after concentration by either ethyl acetate sedimentation or by zinc sulfate floatation.

### Microscopy of wet mount fecal preparations

**Fig. 1:**
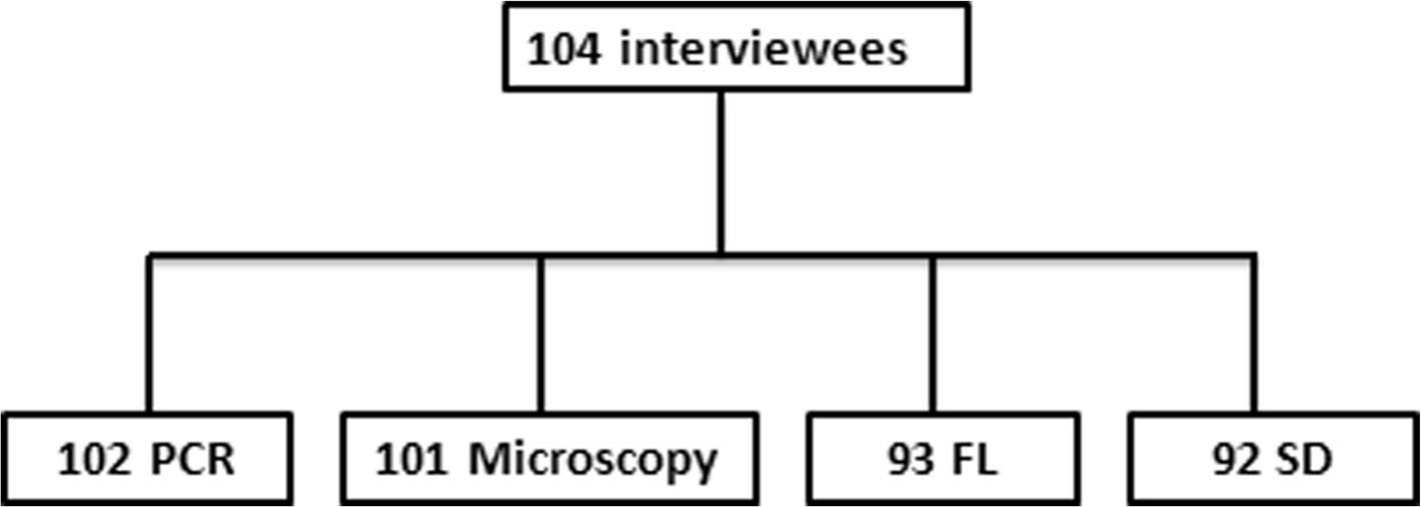
Diagnostic methodology: FL, zinc sulfate floatation; SD, ethyl acetate sedimentation. The discrepancy in numbers of samples is owing to either refraining giving sample following the interview (two individuals) or inadequacy of fecal samples to be tested by all the methods

For each fecal specimen, two wet mount preparations were made on a clean glass slide and under 22x22mm cover slips. For one preparation, the specimen was emulsified with normal saline (0.85% (w/v) NaCl) to enable detection of motile forms of parasites such as trophozoites. For the other preparation, the specimen was emulsified with D’Antoni’s Iodine (1% (w/v) KI and 1.5% (w/v) I_2_, in distilled water) to enable detection of immotile forms of parasites like cysts. The entire area under the cover slip was examined systematically, using a 10x objective, confirming the presence of parasites, using a 40x objective [24]. For quality control and to prevent observer bias, all samples were examined microscopically by two experienced laboratory technicians. One hundred and one samples were examined by microscopy (Fig. 1).

### Fecal concentration methods

Two concentration procedures were used to detect parasites present in small numbers that might have been missed when scanning the wet preparations: ethyl acetate sedimentation and zinc sulphate floatation, as described elsewhere, using the fecal samples preserved in 10% formalin [24]. Ninty- two samples were examined by sedimentation and 93 were examined by floatation (Fig. 1).

### DNA extraction

Genomic DNA was extracted from 0.25 to 0.5 g of fecal specimen, using a Nucleospin® Soil (Machery Nagel GmbH, Düren-Germany) commercial kit with slight modifications that included two pre-treatment steps: the tube containing fecal suspension together with the ceramic beads was incubated at 95 °C for 10 min and was then beaten with a disruptor Genie (Scientific Industries, USA) for 5 min at 2800 rpm before proceeding with manufacturer’s instructions.

### Copro-PCR amplification

PCR primers were selected for the 18S rRNA gene sequences of the species *E. histolytica*, *E. coli*, *E. dispar,* and *E. muris*; GenBank accession numbers AB426549.1 and AB282660.1 for *E. histolytica*; AB445018.1 for *E. muris*, AB282661.1 for *E. dispar*; AF149915.1, AF149914.1, and AB444953.1 for *E. coli*. More versions of the sequence from the same species but of different accession numbers were included to check the stability of the polymorphic regions among the species. The sequences of all the targeted species of *Entamoeba* were aligned, using the Multialin website (http://multalin.toulouse.inra.fr/multalin/) [49]. Primers were designed, using primer 3 on-line software (http://frodo.wi.mit.edu/primer3), in which the conditions were to exclude the polymorphic regions and produce products for the entire sequence (Table 1).

**Table 1:** Primer sets and detection probes for conventional and real-time PCRs used in the study to detect Infections caused by the parasitic species *Giardia lamblia* and *Hymenolepis nana* and those of the genera *Entamoeba* and *Cryptosporidium*

Parasite	Target	Primer	Size, bp	Conditions	Ref.
Conventional PCR					
*E. histolytica*	18S rRNA gene	E-1 5′-GCGGTAATTCCAGCTCCA-3′ E-2 5′-GTGAAATGCTTTCGCTCTCG − 3′	363	95°C5 m [94 °C 30s 56 °C 30s 72 °C 45 s] for 26 × 72 °C 6 m	This study
*E. dispar*			264		
*E. coli*			435		
*E. nana*			645		
*E. muris*			453		
*G. lamblia*	tpi gene ^(1)^	tpi(A-for) 5′-GGAGACCGACGAGCAAAGC-3′ tpi (A-rev), 5′-CTTGCCAAGCGCCTCAA-3′ (Assemblage A)tpi(B-for), 5′-AATAGCAGCACARAACGTGTATCTG-3’tpi(B-rev), 5′-CCCATGTCCAGCAGCATCT-3′(Assemblage B)	184 81	95°C5 m [94 °C 30s 62 °C 30s 72 °C 30s] for 38 × 72 °C 7 m	[26]
*H. nana* *H. dimenuta*	18S rDNA gene, ITS1,	F3 (5′-GCGGAAGGGATACTTACACGTTC-3′) R3 (5-'GCTCGACTCTTCATCGATCCACG-3′)	646 754	95°C5 m [95 °C 20s 60 °C 30s 72 °C 45 s] for 35 × 72 °C 7 m	[27]
*Cryptosporidium spp*	COWP gene^(2)^	cry15: 5′-GTAGATAATGGAAGAGATTGTG-3 cry9: 5′-GGACTGAAATACAGGCATTATCTTG-3’	553	95°C5 m [94 °C 30s 60 °C 30s 72 °C 30s] for 38 × 72 °C 7 m	[28, 29]
Real-time PCR					
*C. parvum spp*	*C. parvum*-specific 452-bp DNA fragment.	Cr-F 5′-CGCTTCTCTAGCCTTTCATGA-3′ Cr-R 5′-CTTCACGTGTGTTTGCCAAT-3′ CryptoTP1 Tex Red- 5′-CCAATCACAGAATCATCAGAATCGACTGGTATC-3′ BHQ2	138	95°C1 5s [95 °C 15 s 60 °C 30s 72 °C 30s] for 40x	[25]
*G. lamblia*	SSU RNA gene ^(3)^	Giardia 80F 5′-GACGGCTCAGGACAACGGTT-3′ Giardia 127R 5′-TTGCCAGCGGTGTCCG-3′ Giardia-105 T FAM-5′-CCCGCGGCGGTCCCTGCTAG-3′-BHQ 1	62	95 °C 15 s [95 °C 15 s 60 °C 30s 72 °C 30s] for 40x	[25]

(1) tpi: triosephosphateisomerase, (2) COWP: *Cryptosporidium* oocyst wall protein, (3) SSU RNA: Small subunit RNA.

The amplification reactions for *Entamoeba spp* considered here were performed in a volume of 25 μl with PCR-Ready™-High Specificity (Syntezza Bioscience Ltd., Jerusalem), 1 μl of 10 μM of each primer, 2 μl of the DNA template and 21 μl nuclease-free water. Positive and negative controls were included. PCRs for the parasites of the species *Giardia lamblia*, *Hymenolpis nana* and *Cryptosporidium spp.* were performed, using the sets of primers and profiles described in Table 1 for the detection and identification of intestinal parasites. Conventional PCRs for the parasites of *Giardia lamblia* and those of the species of *Cryptosporidium spp* were conducted for quantitative real-time PCR (qPCR) to confirm results by subsequent nucleotide Sanger sequencing from both directions followed by a nucleotide BLAST search (https://blast.ncbi.nlm.nih.gov/Blast.cgi?PROGRAM=blastn&PAGE_TYPE=BlastSearch&LINK_LOC=blasthome). Amplification was done, using a T100™ Thermal Cycler **(**Bio-Rad Laboratories, Inc. Hercules, California 94,547, USA). The PCR mix was subjected to the thermal cycling profile given in Table 1. PCR products were separated by electrophoresis at 100 V for 30 min, using a 1.8% agarose gel containing ethidium bromide at a concentration of 0.6 μg/ml (LE Seakam Agarose, Lonza Group Ltd., Muenchen, Steinerstrasse 38 CH-4002, Basel, Switzerland) and Tris-Acetate-EDTA (TAE, pH 8.0) as the running buffer The gel was visualized under a UV viewer with a GeneRuler 100 bp DNA Ladder (Thermo Fisher Scientific, USA) as size marker.

### Copro-PCR, qPCR and conventional PCR

Specific primers and probes were used for amplification as described by Verweij et al. [25]. Standard curves were generated, using pure DNA from parasites of the species *G. lamblia* and those of *Cryptosporidium spp*, by plotting cycle threshold (Ct) values against the log of the DNA concentration of pure samples. To generate the standard curves, the standard DNA sample was adjusted to known concentrations of 5 to 6 points in duplicates in tenfold-serial dilutions at different concentrations of DNA from parasites of the species *G. lamblia* and those of *Cryptosporidium*. Low Ct values corresponded to high amounts of parasite-specific DNA in the samples tested. Samples with Ct above the standard curve were considered positive. qPCR reactions and conditions are given in Table 1.

For inclusion in applying the corpo-PCR, the DNA from the parasites of randomly selected infected cases done by the real-time PCR was amplified by a conventional PCR and the PCR product was DNA sequenced (Hylab, Rehovot) for species identification (Table 1). One hundred and three samples were tested by copro-PCR (Fig. 1).

### Data management and statistical analysis

Data were analyzed, using mainly the EpiInfo statistical package and Prism online calculators. Analysis included distribution, 2 × 2 contingency tables, and frequency Tables. A heat map was constructed based on the bivariate Pearson correlation between types versus number of parasites. Risk predictor variables for parasitic infection were analyzed by the Mid-P exact test as it is less conservative and more powerful than Fisher’s exact test, however, the latter was used whenever a cell was < 5. Chi square and odds ratio with a 95% confidence interval were also calculated and confirmed by multivariate analysis, employing the logistic regression model to calculate the adjusted odds ratio (AOR). Cochrane’s Q, McNemar post hoc test and Dunn’s post hoc test were used to assess performance of the diagnostic methods. The level of statistical significance was *P* < 0.05.

## Results

### Prevalence of intestinal parasites

**Fig. 2:**
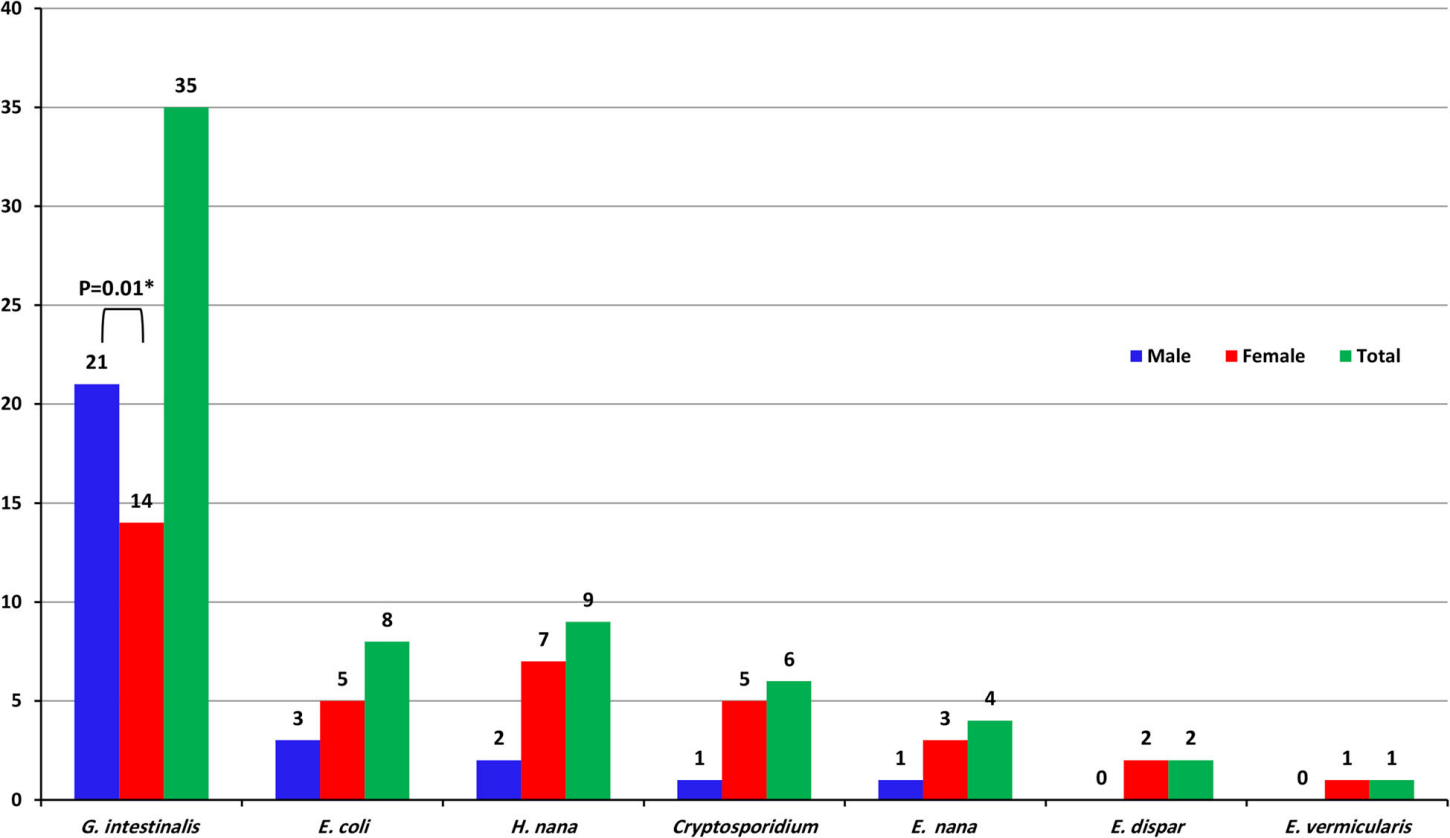
Clustered bar graph, showing the distribution of intestinal parasites detected in the study group according to the sex of group members

**Fig. 3:**
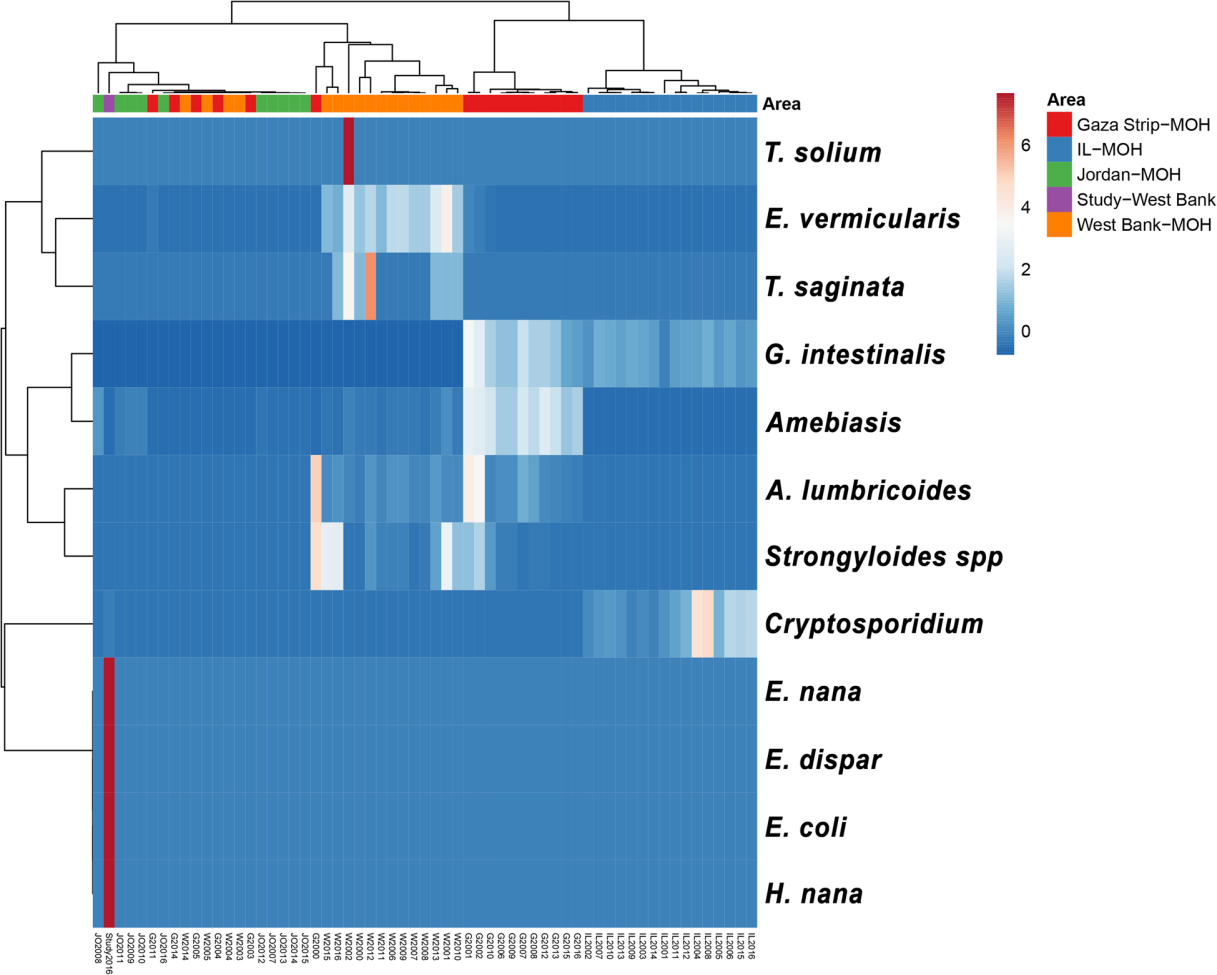
Heat map of the seven types of parasite (*) identified in the study, correlated with the types of parasite reported officially in the region by the ministries of health of the West Bank, the Gaza Strip, Jordan as well as the Israeli Ministry of Health. Rows are mean-centered; unit variance scaling is applied to the rows. Both, rows and columns are clustered, using Pearson correlation distance and average linkage. Each of the 12 rows shows parasite intensity (number). Each column shows the year the parasite was reported during the period 2000 to 2016. Regions coloured red or blue indicate that the number of parasites increased or decreased, respectively

Of the 104 Palestinians studied, 44 (42%) were male and 60 (48%) were female. The median age of the group was 10 years, ranging from 1 to 66 years. The overall prevalence of intestinal parasitic infection was 48% (49/102), 24% (24/102) of which were male. Sixty-seven different parasitic infections were detected among the 49 infected subjects using the four methods described. Thirteen (13%, 13/102) harboured multiple infections, 10% of which were double infections and 3% triple infections. In children less than 14 years old, the infection rate was significantly higher (67%) than in adults (OR = 2.6, *P* = 0.038). To pinpoint a more specific age group, the study group was divided into 4 age groups: 0 to 4 years, 5 to 14 years, 15 to 39 years, and over 40 years. The infection rate was highest in children less than 4 years old, and then gradually decreased with increasing age with border line significance (*P* = 0.05). Seven types of parasitic infection were detected: 37 (37%) infections were caused by the intestinal flagellate *Giardia lamblia*; 11(19%) by the intestinal amoebae, of which 8 (8%) were caused by *Entamoeba coli*, 2 (1%) by *Entamoeba dispar*, 4 (4%) by *Endolimax nana* and 6 (6%) by *Cryptosporidium parvum*; 9 (9%) by the dwarf tapeworm, the cestode *Hymenolepis nana*; 1 (1%) by the nematode *Enterobius vermicularis*. *G. lamblia* was significantly more prevalent among males (*P* = 0.01, Mid-P). The other parasites showed no statistical bias regarding the gender of the examinees (Fig. 2). By applying PCRs, this study revealed the presence of parasites of the species, *E. coli*, *E. dispar*, *E. nana* and *H. nana*, and, for the first time, the absence of *E. histolytica*, based on a review of officially-reported figures (Fig. 3).

### The battery of diagnostic methods

The four types of diagnostic test: direct microscopy of fecal samples in saline with and without iodine; sedimentation, using ethyl acetate; floatation using zinc sulphate; conventional and qPCR were employed to increase sensitivity. Microscopy gave an infection rate of 13% (13/101) and did not expose any of the multiple infections. Of the two concentration procedures used to detect very small numbers of parasites by separating parasites and either their eggs or cysts from fecal debris, ethyl acetate sedimentation was more efficient with a detection rate of 11% (10/92) compared to 7.5% (7/93) by zinc sulphate floatation. Neither of these methods detected multiple infections. Of the methods employed, PCRs amplifying different target genes for different parasites (Table 1) produced the highest infection rate of 45% (46/102), regardless of the type of parasite. Multiple infections were exposed by PCRs with a rate of 13% (13/102). Of these, 10% (10/102) were double infections of *H. nana* and *E. coli* (5/13), of *H. nana* and *G. lamblia* (4/13), of *Cryptosporidium* and *G. lamblia* (1/13) and 3% (3/102) were triple infections. The use of an extended battery of tests increased the detection rate of parasites, however the strength of agreement between these tests was poor (kappa statistic, k = 0.1–0.2) with the exception of the sedimentation and floatation procedures, which was good (k = 0.63). The use of multiple test strategies was further justified as Cochrane’s Q test indicated that there are significant differences between the four methods (Q (3, *N* = 89) = 66.2, *P* < 0.0001). The significance was confirmed by pairwise post-hoc McNemar’s test and pairwise post-hoc Dunn’s test with Bonferroni adjustment), which revealed high variation when the PCR methods were included in the comparison (*P* < 0.0001) (Table 2). Of the 37 cases caused by *G. lamblia*, PCRs detected 24 (73%) that were not detected by the conventional methods with only two misses in favour of the conventional methods.

**Table 2:** Results of the four diagnostic methods employed. The term PCR covers and combines the results of conventional PCRs and real time qPCRs

	Sedimentation				PCR			Floatation		
		+	–	∑	+	–	∑	+	–	∑
Microscopy	+	1	2	3	12	0	12	2	3	5
	–	9	80	89	33	57	90	5	83	88
	∑	10	82	92	45	57	102	7	86	93
Floatation	+	4	1	5	4	2	6			
	–	4	81	85	31	55	86			
	∑	8	82	90	35	57	92			
PCR	+	8	27	35						
	–	2	55	57						
	∑	10	82	92						

### DNA sequencing and species identification

The methods employing PCRs exposed 46 cases, which, collectively, harboured 62 different identified parasitic infections. Of these, 27% (17/62) were successfully sequenced and identified further by applying BLAST. The comparison of the PCR-amplified sequences from the positive cases showed 97–99% sequence identity and 100% coverage when aligned against the reference sequences of the species *E. coli*, *H. nana, E. dispar,* and *Cryptosoriduim*. Representative nucleotide sequences generated in this study were deposited in the GenBank under the accession numbers shown in Table 3, except for two sequences from parasites of the species *G. lamblia* that were less than 200 bp, the minimum number of nucleotides processed by the GenBank. DNA sample from parasites of the genus *E. vermicularis* were not included in the molecular diagnostic panel and the parasites were only confirmed microscopically. Randomly selected DNA samples from parasites of the species *E. nana* and *C. parvum* were sequenced successfully but at a low percentage of identity compared to the Gene Bank reference strain.

**Table 3:** The parasites detected by all four diagnostic tests, giving their Accession Numbers for the GenBank

	PCR		WM	SD	FL	Overall*
Parasitic species	Positive (%)	Acc. No.	Positive (%)	Positive (%)	Positive (%)	Positive (%)
*G. lamblia*	34 (34)	–	6 (6)	5(6)	3 (3)	37 (36)
*E. coli*	7 (7)	MH629959, MH629962–6	2 (2)	3 (3)	2 (2)	8 (8)
*H. nana*	8 (8)	MH629967–73	4 (4)	1 (19	0 (0)	9 (9)
*Cryptosporidium*	6 (6)	–	0 (0)	0 (0)	0 (0)	6(6)
*E. dispar*	2 (2)	MH629960–1	0 (0)	1 (1)	1 (1)	2(2)
*E. nana*	4 (4)	–	0 (0)	0 (0)	0 (0)	4(4)
*E. vermicularis*	- (−)	–	1 (1)	0 (0)	1 (1)	1(1)
	61/102 (60)		13/101 (13)	10/92 (11)	7/93 (8%)	67/102 (66)

Key: PCR, conventional and real-time polymerase chain reactions; Acc. No., accession number at GenBank; WM, wet mount preparation microscopy; SD, sedimentation method; FL, floatation method. * Based on at least one positive result by at least one method without repetition in cases detected by more than one method giving 61 parasitic infections of 7 different species in 46 individuals.

### Risk predictors for intestinal parasitic infection

Analysis of the three domains of risk assessment: demography; socioeconomic status (SES); hygiene and behavior showed young age (< 14 yrs) (OR = 2.61, P = 0.038) is a risk factor for acquiring intestinal parasitic infections (Table 4). The potential risk factors as independent predictor variables were analyzed statistically against each separate parasitic infection as dependent outcomes. No significant results were obtained except for the gender of participants as a risk factor for infections caused by parasites of the species *G. lambila* (OR = 3.5, *P* = 0.0054). The other risk predictor was the source of drinking water with regard to infections caused by parasites of the species *H. nana* (OR = 14.16, *P* = 0.0006), which confirmed the borderline significance found in the overall infection with intestinal parasites. The adjusted odds ratio (AOR) showed a significant difference in the infection rate among families of low income (< 2000 per month), however, the small sample size may have widened the 95% confidence interval, which could negatively affect precision. An AOR showed that low monthly income, which is a reflection of SES, is a risk factor (AOR = 22.7, *P* = 0.023).

**Table 4:** The major domains of risk factors associated with the study group acquiring parasitic infections: demography; socioeconomic status (SES); hygiene and behavior

Test								
Variable	Category	Positive	Negative	Total	COR (CI 95%)	*P*-value	AOR (CI 95%)	*P*-value
Demography								
Sex	Male	24	20	44	1.8 (0.82–3.95)	0.07	0.42(0.15–1.22)	0.11
	Female	24	36	60				
	Total	48	56	104				
Age	< 14 yrs	32	27	59	2.61(1.05–6.45)*	0.038*	0.36(0.11–1.12)	0.07
	> 14 yrs	10	22	32				
	Total	42	49	91				
Size of family	< 7	2	7	9	0.3 (0.06–1.5)	0.15	1.37(0.15–11.8)	0.77
	≥7	41	43	84				
	Total	34	50	101				
SES								
Level of education, HOH	< 12 yrs	35	42	77	0.83 (0.33–2.09)	0.35	0.71(0.09–2.51)	0.75
	> 12 yrs	12	12	24				
	Total	47	54	101				
Level of education, mother	< 12 yrs	19	68	87	0.85 (0.28–2.63)	0.39	0.51(0.03–6.76)	0.61
	> 12 yrs	2	12	14				
	Total	21	80	101				
Monthly income	< 2000	26	45	71	0.47 (0.17–1.29)	0.16	22.7(1.5–337)	0.023**
	> 2000	11	9	20				
	Total	37	54	91				
Meat consumption	<= 1 /wks	19	21	40	1.07 (0.48–2.37)	0.44	0.74(0.15–3.57)	0.71
	2 > 1 /wks	28	33	61				
	TOTAL	47	54	101				
Hygiene and Behavior								
Toilette, type	Pit	30	29	59	1.52 (0.68–3.39)	0.16	0.73(0.13–4.1)	0.72
	Flush	17	25	42				
	TOTAL	47	54	101				
Wash hand before meal	Always	12	13	25	1.21 (0.48–3.04)	0.35	–	–
	Sometimes	29	38	67				
	Total	41	51	92				
Wash hands after toilette	Always	17	17	34	1.42 (0.60–3.32)	0.22	–	–
	Sometimes	24	34	58				
	Total	41	51	92				
Rats/mice in house	Yes	23	23	46	1.35 (0.61–2.97)	0.23	1.23(0.29–5)	0.77
	No	23	31	54				
	Total	46	54	100				
Source of drinking water	tap in house	39	51	90	0.27 (0.07–1.15)	0.08	0.17(0.01–2.1)	0.18
	tap in yard	8	3	11				
	Total	47	54	101				
Beatles in house	Yes	38	71	79	1.33 (0.51–3.49)	0.28	0.56(0.34–126)	0.21
	No	9	13	22				
	Total	47	54	101				
Dirty nails	Yes	17	21	38	0.91 (0.41–2.03)	0.42	0.30(0.03–2.5)	0.27
	No	31	35	66				
	Total	48	56	104				
Wear shoes in yard	Yes	8	6	14	1.93 (0.59–6.37)	0.15	–	–
	No	22	32	54				
	Total	30	38	68				
Eat raw vegetables	Always	45	53	98	0.87 (0.04–4.84)	0.45	0.55(0.01–18)	0.74
	Sometimes	2	1	3				
	Total	47	54	101				

* Significant by crude odds ratio (COR) with Mid-P test at *P* value ≤0.05 ** significant by adjusted odds ratio (AOR) with multivariate logistic regression model at P-value ≤0.05: HOH, Head of Household; SES, Socioeconomic Status; COR, Crude Odds Ratio; AOR: Adjusted Odds Ratio

## Discussion

The prevalence of intestinal parasitic infections in the population studied, which lived in an under-privileged rural area in the Palestinian part of the Jordan Valley, was 48%. This was considered high compared to a prevalence of 20.2% found in school children in the rural and urban areas of the northern part of Palestine a decade ago and even to countries of high endemicity [8, 22, 30]. The rate of intestinal parasitic infections among the Palestinian public in Palestine during the British Mandate declined from 86% in the 1940s [6] to about 70% in the1980s [9] but remained high as this study showed (46%). Some of the parasites reported earlier, *Trichuris trichura, Hymenolepis nana,* and *Ancylostoma duodenale*, have, according to official reports and studies, disappeared from the West Bank. The high prevalence could be owing to poor personal hygiene [22] and improper agricultural and commodity marketing practices such as using night soil as fertilizer and moistening fruits and vegetables with contaminated water to attract customers. Furthermore, livestock, cows and sheep, is kept in and around the houses and dung on the sides of streets attracts flies, cockroaches and beetles. The Jewish minority in Palestine during the 1930s and 1940s also suffered from a high prevalence of intestinal parasitic infections such as *Ascaris lumbricoides* (40%) which may have been shared between the two communities or brought with immigrants from Europe after the World War II [31]. This study showed that *H. nana* is still circulating (9%) but the parasitical epidemiology has changed with the loss of *A. lumbricoides,* and *E. histolytica,* and *G. lamblia,* and *H. nana* persisting as the leading causes of infection, demonstrating changing patterns of intestinal parasitic infections over time. Similar studies were conducted in the Gaza Strip that showed rates ranging from 28 to 53% [10–12, 32]. In the Gaza Strip, the Ministry of Health still reports high incidence rates that reached 407 per 100,000 in 2016 with an actual total of 7651 cases, which were mainly cases of amoebiasis and giardiasis (Fig. 3). However, a study in the Gaza Strip revealed rare parasitic infections caused by *H. nana* and *Trichuris trichura* [32]. In the West Bank, the incidence rate of parasitic infections in the same year was 69 per 100,000 with an actual total of 1881 infections [13]. Official reports in 2016 put the incidence rate among Israeli citizens at 17 per 100,000 with an actual total of 1370 infections, which were mainly restricted to infections caused by the species *G. lamblia* with several dozen caused by species of *Cryptosporidium* [14, 33]. A study on parasitic infections reported a prevalence of 26% among the Israeli pediatric population, 36% among Palestinian Bedouins, and 11% among the Jewish community with parasitic infections being caused by *Entamoeba spp*, *Blastocystis hominis*, *G. lamblia*, *E. vermicularis*, and rarely *H. nana* [34]. On the other hand, prevalence was only 10 per 100,000 in neighboring Jordan and was restricted only to amoebiasis [15]. A more comprehensive and thorough study conducted in the north of Jordan revealed a higher rate of 44% with an approximate incidence rate of 148 per 100,000 caused by seven parasitic species and like our study showed the species *G. lamblia* to be the most prevalent parasite with rare (< 1%) infections caused by the species *H. nana* and *Chilomastix mesnili* [35]. Multiple intestinal parasitic infections increase the burden of disease and were recorded at a rate of 46% in Palestine of the 1940s*.* Seventy years later, this has become 13% in the West Bank as revealed by this study and 9% in the Gaza Strip, which is considered high compared to 3% in an Ethiopian endemic rural area [6, 12, 30]. The political situation in this region might have impacted negatively on the prevalence of intestinal parasitic infections, in the 1930s and 1940s, owing to neglect during British rule and ending with a war in 1948. The neglect, crowdedness, poverty, the collapse of the infrastructure, resulting in sewage pouring into the sea and wars could explain the high prevalence of parasitic infections in the Palestinian population. Also, imported intestinal parasitic infections have been reported in the region through mass immigrations as in the case of the Falasha Jews from Ethiopia and an imported Thai labour force, including Thai workers suffering from parasitic infections new to the region such as those caused by trematodes (flukes) [36, 37].

The efficiency of the surveillance system is crucial in assessing the credibility of government reports and is to a large extent affected by the method chosen for diagnosis. Ministry of Health laboratories in Palestine in the West Bank and Gaza Strip use microscopy of wet mount preparations made with normal saline as the sole method of diagnosis, which seriously underestimates the real prevalence and burden of infection. However, misdiagnosis cannot be ruled out, considering the absence of a Palestinian parasitology reference laboratory and the heavy workload in Palestinian government laboratories. This makes taking the time to microscopically scan whole fecal preparations with few parasites or using other diagnostic methods on them unlikely. In addition, misdiagnosis of leukocytes as cysts of parasites of the various *Entamoeba spp* is common. Factors like these have led to Jordanian diagnoses of intestinal parasitic infections being reported only as amoebiasis and Palestinian reports certifying cases to be amoebiasis without identifying and stating the species, *i*. *e*. *E. histolytica*, *E. dispar*, *E. moshkovskii*, or *E. coli*. Amplifying the 18S rRNA gene of the parasites causing the cases of amoebiasis diagnosed in this study enabled their detection and their identification to the species level. The panel of the four diagnostic methods used did not agree (k < 0.2) fully in terms of overall results except for floatation and sedimentation, which agreed 94% of the time (Kappa = 0.6). Thus, ethyl acetate sedimentation could be used as an alternative to zinc sulfate floatation, albeit sacrificing 1% of the positive cases. Furthermore, statistical analysis revealed insignificant differences in diagnostic performance between methods, except when PCRs were applied, which enable the detection of a part of a parasite of just a few DNA copies, a size which can never be detected by light microscopy and also has the advantage of enabling species identification [38]. Despite employing PCRs, parasites of the pathogenic species *E. histolytica* and the possibly pathogenic species *E. moshkovskii* were not detected in fecal samples from examined individuals but parasites of the non-pathogenic species *E. dispar* were. This is of paramount importance, knowing that the three species are morphologically indistinguishable but with the pathogenic species *E. histolytica* generally constituting 10% of the three types. These findings should change disease management and drug prescription. Moreover, the use of PCRs led to the exposure of parasites of the species *E. nana* in the human population of the West Bank for the first time and their high sensitivity increased detection of giardiasis from 13% by the conventional methods to 37% by applying qPCRs. This agrees with other studies [38–41]. As revealed here, the introduction of PCRs enabled detection of multiple infections of either two or three different types of parasite.

Age is an important risk predictor for intestinal parasitic infections. This study showed that children under 14 years old were more prone to infection than people over 14 years old (OR = 2.6, *P* = 0.038). This could be owing to immaturity of the immune system, more time spent on outdoor activity and curiosity leading to exploring surroundings without adhering to hygiene. The effect is greater when the surrounding environment lacks infrastructure such as intact sewage systems, clean water, exposure to waste owing to unclean streets and inadequate dumping facilities. The effect of age has been reported following studies done in Ethiopia, Jordan and Qatar [20, 30, 35, 42]. This study showed that the chances of males contracting infections are 1.8 times higher compared to females but marginally insignificant (*P* = 0.07, CI: 0.82–3.95). This agrees with some of the studies cited just above [20, 30, 35]. This study indicated that age had no effect on infections caused by parasites of the species *G. lamblia, E. nana* and *E. coli* but those of *G. lamblia* were affected by hosts’ gender as also reported elsewhere [30, 42]*.* Low-income families, subsequently classified as low socioeconomic status (SES), are significantly exposed to parasitic infections, which can be a direct effect of malnutrition as shown in other studies [43]. A study in Guinea-Bissau showed that water supply was a risk factor in acquiring parasitic infections, which paralleled the findings of this study but only in the case of infections caused by *H. nana* [22].

A limitation of this study was the use of a cross-sectional design for risk assessment with a focus on intra-village comparison. The ideal design would have been an incident case-control study focusing on both intra- and inter-village comparisons. In light of official reports, the PCR-based tests should have been applied to other parasitic helminths, cestode species of the genus *Taenia* and nematode species of the genus *Ascaris*, *Enterobius*, and *Trichuris.* Also, the sample size was small, which can affect statistical inference.

## Conclusion

The prevalence of intestinal parasitic infections in marginalized rural areas in the Palestine in the West Bank Region remains high with parasites of the species *G. lamblia* and *H. nana* being their main cause. Parasites of the species *E. histolytica*, the main pathogen causing severe amoebiasis, are absent. PCR-based methods should be considered essential in the diagnosis of intestinal parasitic infections and identification of the parasites causing them owing to their high sensitivity. The Palestinian Ministry of Health should establish parasitology diagnostic facilities in the West Bank and the Gaza Strip that employ various diagnostic methods, including molecular-based methods to overcome underestimation of incidence rates, avoid misdiagnosis, and help in disease management and drug prescription.

## Data Availability

The data used and analyzed during this study are available from the corresponding author on reasonable request.

## Abbreviations

COWP: *Cryptosporidium* oocyst wall protein
DNA: deoxyribonucleic acid
GBD: global burden of disease
HOH: head of household
OR: odds ratio
PCR: polymerase chain reaction
qPCR: real-time polymerase chain reaction
RNA: ribonucleic acid
SES: socioeconomic status
SSU RNA: Small subunit RNA
tpi: triosephosphateisomerase
WHO: world health organization
